# The Prognostic Role of Polysomnography Parameters in Heart Failure Patients with Previous Decompensation

**DOI:** 10.3390/jcm11133656

**Published:** 2022-06-24

**Authors:** Elena Medvedeva, Lyudmila Korostovtseva, Mihail Bochkarev, Anastasiya Shumeiko, Aelita Berezina, Maria Simonenko, Yulia Sazonova, Andrey Kozlenok, Yurii Sviryaev

**Affiliations:** Almazov National Medical Research Centre, 197341 St. Petersburg, Russia; lyudmila_korosto@mail.ru (L.K.); michail_bv@list.ru (M.B.); nastya0594@mail.ru (A.S.); berezina_av@almazovcentre.ru (A.B.); ladymaria.dr@gmail.com (M.S.); yulia.via.sazonova@gmail.com (Y.S.); kozlenok_av@almazovcentre.ru (A.K.); yusvyr@yandex.ru (Y.S.)

**Keywords:** heart failure, sleep apnea, prognosis, polysomnography, wake after sleep onset

## Abstract

Background: Sleep-disordered breathing (SDB) is a widespread comorbidity in patients with chronic heart failure (HF) and may have a deleterious effect on the pathogenesis of HF. We aimed to evaluate the prognostic role of polysomnography parameters in HF patients with previous decompensation. Methods: 123 patients were included in the prospective cohort study. In addition to the standard examination, all patients underwent polysomnography (PSG). Results: The Kaplan–Meier analysis showed the incidence of the combined endpoint differs between LVEF categories ≤25.5% vs. >25.5% (χ^2^ = 9.6, log rank *p* = 0.002), NTpro-BNP > 680 vs. ≤680 pg/mL (χ^2^ = 12.7, log rank *p* = 0.001), VO_2_peak categories <16 vs. ≥16 mL/min/kg (χ^2^ = 14.2, log rank *p* = 0.001), VE/VCO_2_ slope ≥38.5 vs. <38.5 (χ^2^ = 14.5, log rank *p* = 0.001), wake after sleep onset >40 min vs. ≤40 min (χ^2^ = 9.7, log rank *p* = 0.03), and sleep stage 2 (S_2_) <44% vs. ≥44% (χ^2^ = 12.4, log rank *p* = 0.001). Conclusion: Among the PSG parameters, WASO > 40 min and S_2_ < 44% were associated with a combined endpoint in patients with previous decompensation of HF. Moreover, higher NT-proBNP and VE/VCO_2_ slope, lower LVEF, and VO_2peak_ were also independent factors of a poor prognosis.

## 1. Introduction

Sleep-disordered breathing (SDB) is associated with more frequent readmissions and worse outcomes in patients with heart failure (HF) [1,2,3]. According to several studies, sleep apnea in patients with HF is associated with an increased frequency of life-threatening arrhythmias during night hours, confirmed by an increased frequency of nocturnal shocks from implantable cardioverter–defibrillators [4].

While obstructive sleep apnea (OSA) is shown to be an independent risk factor for the development of HF and a worse prognosis [5], central sleep apnea (CSA) is possibly a marker of HF severity, with increased neurohumoral activation, increased pulmonary capillary wedge pressure, and worsening HF [6]. The sympathetic activation that occurs in patients with CSA might adversely affect the prognosis in HF patients. The sympathetic activation is associated with the incidence of apneas, frequency, and severity of hypoxia through chemoreflex activation. This mechanism suggests that CSA can play a crucial role in the worsening of the left ventricular function and clinical status of HF patients, emphasizing the existence of a bidirectional relationship between CSA and HF [1].

The severity of apnea is usually assessed using the apnea–hypopnea index, but this index, associated with prognosis, probably does not reflect all the complex pathophysiological mechanisms that can lead to adverse outcomes in heart failure. Moreover, the prognostic value of the parameters characterizing the structure and quality of sleep in HF patients is still uncertain.

Episodes of decompensation of HF worsen the prognosis and exacerbate comorbid conditions. In the present study, we aim to evaluate the clinical features and the prognostic role of polysomnography parameters in HF patients with previous decompensation.

## 2. Materials and Methods

### 2.1. Patients and Study Design

In a prospective cohort study, we enrolled patients who were eligible according to the following inclusion criteria: patients with chronic heart failure II-IV functional class NYHA, 18–74 years old, and hospitalized with decompensation of HF.

Exclusion criteria were inability to participate or consent, acute coronary syndrome or recent stroke (within 90 days of admission), severe chronic obstructive pulmonary disease (COPD) with forced expiratory volume in 1 s (FEV1) less than 50%, and oxygen therapy.

All patients underwent standard clinical examination after compensation, a 6 min walking test, 12-lead electrocardiography, transthoracic echocardiography, polysomnography, cardiopulmonary exercise testing, and venous blood sampling. All examinations were performed before discharge in patients without significant congestion, if possible, and while sleeping in a horizontal position. The main characteristics of the patients are presented in Table 1.

The study protocol was approved by the ethics committee. All participants signed written informed consent.

### 2.2. Methods

#### 2.2.1. Polysomnography

After compensation, the patients underwent in-lab polysomnography (PSG) (Embla N7000, Natus, Middleton, WI, USA). If patients needed constant monitoring, a portable recording system was used to perform PSG in heart failure units (MiniScreen Pro, Löwenstein Medical, Hamburg, Germany). PSG was repeated in patients who underwent heart transplantation (HTx) during follow-up (usually 6 months after transplantation).

PSG analysis was manually performed by two experienced investigators using custom software. Episodes of apnea–hypopnea were assessed according to the guidelines of the American Academy of Sleep Medicine Task Force [7]. Based on the apnea–hypopnea index (AHI), sleep apnea was defined as none (AHI < 5/h), mild (AHI 5–14.9/h), moderate (AHI 15–29.9/h), or severe (AHI ≥ 30/h). We also estimated the following indicators: total sleep time (TST), duration of sleep stages (S_1_, S_2_, S_3_, REM), WASO (wake after sleep onset), sleep efficiency, oxygen desaturation index (ODI), average and minimum saturation O_2_, and the maximum and mean duration of apnea.

#### 2.2.2. Echocardiographic Examination

All patients underwent complete transthoracic echocardiographic studies with a Vivid 7 (GE Vingmed Ultrasound AS, Horten, Norway) system. Two-dimensional-mode (2Dmode), M-mode echocardiography, and tissue Doppler imaging were conducted. Three consecutive cycles were averaged for every parameter. The guidelines and expert consensus for echocardiographic cardiac chamber quantification were used for further imaging analysis [8,9].

#### 2.2.3. Cardiopulmonary Exercise Testing

Cardiopulmonary exercise testing (CPET) was conducted using the breath-by-breath method on an ergospirometer, Oxycon Pro (Cardinal Health, Norderstedt, Germany). Patients performed symptom-limited, continuously increasing physical activity (RAMP protocol) on a bicycle ergometer with a load increment of 10 W/min. The subjective sensations of the patients were evaluated on a 10-point Borg scale.

Criteria for test termination consisted of the following: achievement of the maximum possible physical load for the individual or listed conditions, significant chest pain, ischemic changes in the ECG (ST segment depression > 2.0 mm; T wave inversion; new Q wave), decrease in blood pressure, sustained supraventricular or ventricular tachycardia, presyncope, syncope, unbearable dyspnea, disorientation, and loss of coordination. 

The following test parameters were evaluated: peak oxygen consumption (VO_2_peak), anaerobic threshold level, heart rate increase (HR) during testing (HR/VO_2_ slope), HR reserve (the difference between the predicted HR max and HR max achieved during testing), the oxygen pulse (VO_2_/HR), and the ventilator equivalent of CO_2_ (VE/VCO_2_ slope). The value of VO_2_peak or VO_2_max ≥ 85% of the proper value and the level of anaerobic threshold corresponding to 40–60% of VO_2_max were considered normal.

#### 2.2.4. Blood Analysis and BIOMARKERS

Blood samples were collected one week after compensation. The following blood tests were conducted: complete blood count, electrolytes, blood chemistry, lipid profile values, and serum protein. The glomerular filtration rate (GFR) was calculated by the CKD-EPI equation.

The plasma levels of the N-terminal prohormone of brain natriuretic peptide (NT-proBNP) and soluble form of suppression of tumorigenicity-2 (sST2) were measured after compensation using the enzyme immunoassay technique (ELISA kit), according to the instructions of the manufacturer.

#### 2.2.5. Follow-Up

The individuals were followed up with for a maximum of 9.14 years. The collection of information about adverse events was carried out twice a year and provided by patients via telephone calls. Other status reports and information about the time and cause of death were obtained from various sources, including relatives, general practitioners, hospitals, and local registration offices.

The combined endpoint included the first event of death from any cause, left ventricular assistant device (LVAD) implantation, HTx, non-fatal myocardial infarction, repeat revascularization, and stroke. Death was considered a priority event, and the time of occurrence of the event was regarded as the time of death.

During follow-up, patient management was conducted according to discretion of the treating physician based on the European Society of Cardiology guidelines.

#### 2.2.6. Statistical Methods

Continuous variables were presented as mean± standard deviation or median (quartile 1–quartile 3) for skewed distributions. Categorical variables were mentioned as frequencies in percent. For testing the hypotheses, 2-sided alternatives were analyzed, and the type I error rate was 5%. Due to the abnormality of the distribution, the Mann–Whitney test was used to compare independent samples, and Fisher’s exact test was used for comparing categorical variables. 

To investigate the predictive abilities of different parameters, a ROC curve analysis was constructed. Survival analysis was carried out using Kaplan–Meier curves. Log rank tests were used to compare survival between the different groups. All analyses were conducted in IBM SPSS Statistics version 25.0.

## 3. Results

### 3.1. HF Population

All patient characteristics are described in Table 1. Coronary artery disease was present in 58.7% of patients. The median of left ventricular ejection fraction (LVEF) was 27.0%, and only 8.3% of patients had HF with preserved EF.

### 3.2. Prognosis

According to follow-up data, 76 patients reached the combined endpoint. The distribution by clinical, laboratorial, and instrumental parameters is presented in Table 2.

Compared to the group without a combined endpoint, the group with adverse outcomes had higher NYHA classes, a lower LVEF, a higher NT-proBNP, a lower peak oxygen consumption, and a higher ventilator equivalent of CO_2_. In patients with adverse events, AHI, SpO2, and ODI were comparable to patients without events. However, event patients had a significantly longer WASO time and shorter duration of S_2_ (Table 2).

**Figure 1:**
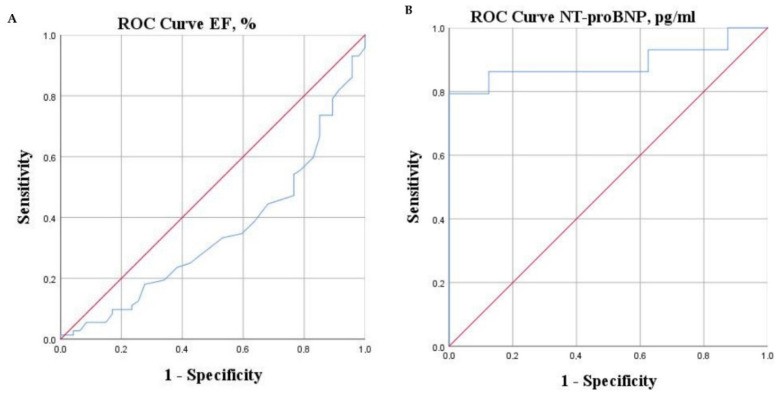
ROC curves of LVEF (**A**) and NT-pro-BNP (**B**).

**Figure 2:**
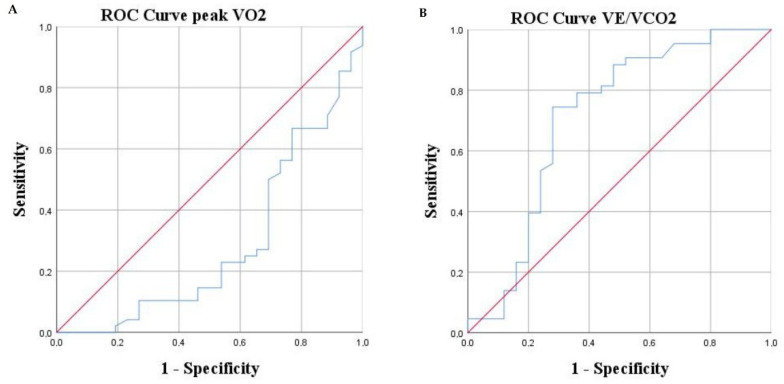
ROC curves of CPET parameters: V0_2_peak (**A**), VE/VCO_2_ (**B**).

**Figure 3:**
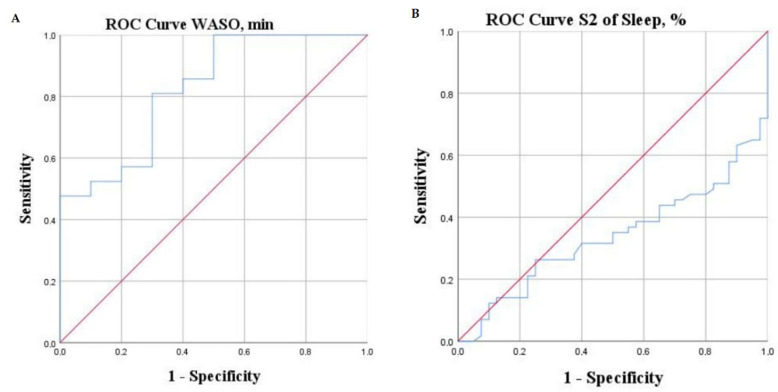
ROC curves of PSG parameters: WASO (**A**), S_2_ (**B**).

To investigate the predictive abilities of different parameters, a ROC curve analysis was constructed (Table 3 and Figure 1, Figure 2 and Figure 3).

We analyzed cut-offs of identified significant parameters that would better identify patients at high risk of adverse outcomes. A significantly higher risk of adverse events was observed in patients with LVEF ≤ 25.5%, NT-proBNP > 680 pg/mL, VO_2peak_ < 16 mL/kg/min, VE/VCO_2_ slope ≥ 38.5, S_2_ <44%, and WASO > 40 min.

**Figure 4:**
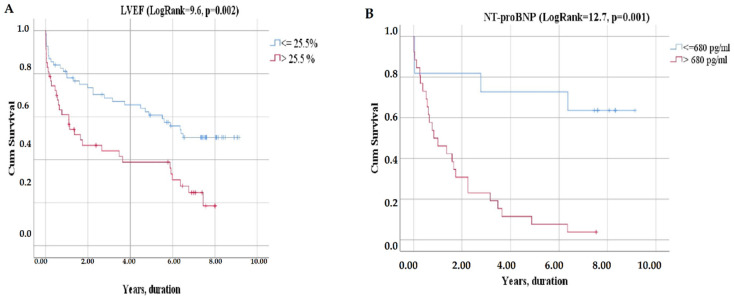
Kaplan–Meier curves of event-free survival according LVEF (**A**), NT-proBNP (**B**).

**Figure 5:**
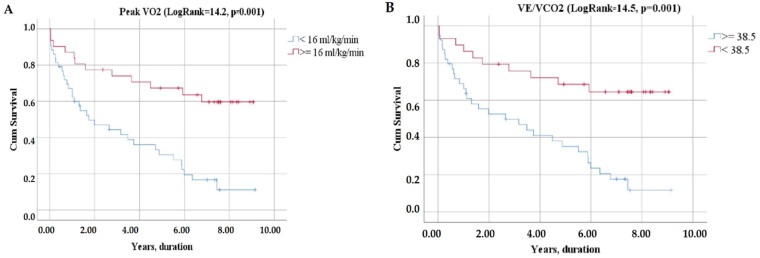
Kaplan–Meier curves of event-free survival according to CPET parameters: V0_2_peak (**A**), VE/VCO_2_ (**B**).

**Figure 6:**
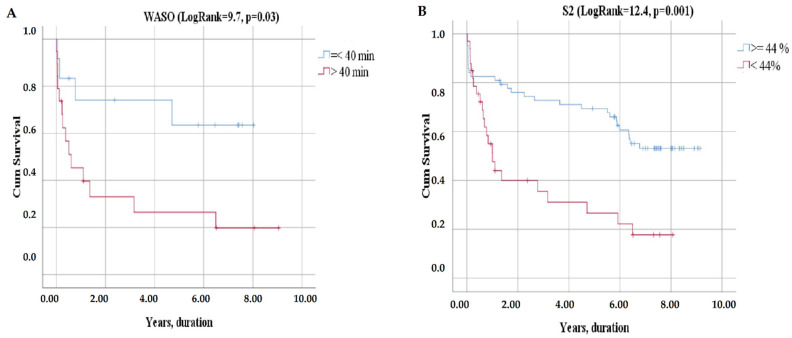
Kaplan–Meier curves of event-free survival according to PSG parameters: WASO (**A**), S_2_ (**B**).

The Kaplan–Meier analysis demonstrated that the combined endpoint significantly differs between LVEF categories, NTpro-BNP categories (Figure 4), VO_2_peak categories, VE/VCO_2_ slope categories (Figure 5), WASO categories, and S_2_ categories (Figure 6).

The proportion of patients surviving 8 years was significantly lower in patients with LVEF ≤ 25.50% compared to LVEF > 25.5%—0% vs. 19% (χ^2^ = 9.6, log rank *p* = 0.002) (Figure 4A).

There was also an observed difference in survival between HF patients with NT-proBNP level > 680 pg/mL and those with an NT-proBNP level ≤ 680 pg/mL (χ^2^ = 12.7, log rank *p* = 0.001): eight-year survival was 4% vs. 36%, respectively (Figure 4B).

Eight-year survival was 2% for those with VO_2_peak < 16 mL/kg/min, 23% for those with VO_2_peak ≥ 16 mL/kg/min, 3% for patients with VE/VCO_2_ slope ≥ 38.5, and 24% for those below this cut-off (Figure 5).

The patients with WASO > 40 min and S_2_ <44% had 8-year survival rates of 5% and 3% compared to 8% and 19% for those with WASO ≤40 min and S_2_ ≥ 44%, respectively (Figure 6).

## 4. Discussion

According to the present study, patients with adverse events had a comparable AHI to those without an endpoint (*p* = 0.9). The limited role of AHI in HF is noted by many authors [10,11]. The severity of apnea, and most importantly its effect on outcomes, may be determined not only by AHI. This problem is discussed in the literature, and research is being conducted in this direction [12]. B. Gellen et al., demonstrated that the nocturnal desaturation is a marker of poor prognosis and helps identify high-risk patients. Moreover, severe nocturnal desaturation is associated with a poor prognosis, regardless of the existence of significant sleep apnea [13]. The authors described four different phenotypes depending on the presence of sleep apnea and minimal nocturnal oxygen saturation during sleep ≤ 88%.

Y. Huang et al., in a prospective cohort study, also demonstrated the prognostic role of nocturnal hypoxemia in patients with HF. A multivariate Cox regression analysis found that a saturation below 90% was independently associated with the outcome (hazard ratio [HR] 1.008, 95% confidence interval [CI] 1.001–1.016, *p* = 0.033), while no such association was found for AHI [11]. However, in the present study, the group with adverse events and the group without a combined endpoint had a comparable SpO_2ave_ (*p* = 0.4), SpO_2min_ (*p* = 0.5), ODI (*p* = 0.3), according to ROC analysis. AUC for SpO_2_ was 0.55 (95%CI 0.439–0.661, *p* = 0.057). This can be explained by different designs and methods. In their study, Huang Y. et al., included patients with acute decompensated HF and used a portable screening device (class 4 equipment with certain limitations) rather than full PSG, which is explained by the clinical status of patients. In our study, patients showed no evidence of fluid overload and/or hypoperfusion at the time of PSG, in contrast to Huang’s study. Such symptoms are associated with severe congestion in the lungs and hypoxia; therefore, in our opinion, it is difficult to identify the contribution of apnea to the development of hypoxia in patients with acute decompensation. Moreover, as previously stated, conducting a full PSG allowed us to obtain more data and include more variables in the analytical matrix.

Our results demonstrate that assessing the prognostic role of polysomnography indicators is a multicomponent problem, and a heterogeneous cohort of patients with heart failure with previous decompensation may have different PSG predictors of poor prognosis: WASO and duration of S_2_. The current study also found other laboratorial and instrumental parameters (NT-proBNP, VO_2_peak, VE/VCO_2_ slope) that were independent risk factors for a combined endpoint.

B. Yan et al., in a community-based cohort study (*n* = 3810), found that sleep efficiency and wake after sleep onset were predictors of major adverse cardiovascular events. Wake after sleep onset >78minutes was associated with primary (HR, 1.436; 95% CI, 1.066–1.934; *p* = 0.017) and secondary composite cardiovascular outcomes (HR, 1.374; 95% CI, 1.103–1.712; *p* = 0.005), as well as cardiovascular mortality (HR, 2.240; 95% CI, 1.377–3.642; *p* = 0.001) [14]. We also established the significant prognostic role of WASO, but with another cut-off level of >40 min, due to examination of a completely different cohort of patients. On the one hand, such an association can be explained by the presence of severe symptoms of HF in patients at night; on the other hand, long WASO may contribute to the progression of HF due to the activation of the sympathetic–adrenal system and other pathogenetic factors (endothelial damage and dysfunction, progression of atherosclerosis, etc.) [15].

The present study has the following limitations: first of all, a relatively small sample size can reduce the generalizability of the predictive value of PSG and other parameters. The studied cohort is heterogeneous in terms of LVEF, functional classes, and the etiology of HF. However, we recruited patients who were stabilized after previous decompensation of HF and aimed to identify the role of PSG indicators in prognosis.

Thus, according to our results, we can conclude that WASO > 40 min and S_2_ <44% were independent predictors of a combined endpoint in patients with previous decompensation of HF. These PSG parameters may be used for estimation prognosis as well as NT-proBNP, LVEF, VE/VCO_2_ slope, and VO_2peak_.

## Figures and Tables

**Table 1:** Baseline characteristics of the cohort.

Parameter	Baseline
**Age, years**	55 (46–60)
**Male/female, *n***	103/20
**Body mass index, kg/m^2^**	26.13 (22.2–32.75)
**NYHA functional class, *n***	
II	46
III	52
IV	25
**Left ventricle ejection fraction (LVEF), %**	27 (21–32.5)
Reduced LVEF (<40%), *n*	108
Midrange LVEF (40–49%), *n*	10
Preserved LVEF (>50%), *n*	5
**HF etiology**	***n***
Coronary artery disease (CAD), *n*	73
Non-CAD, *n*	50
*Dilated cardiomyopathy*	44
*Restrictive cardiomyopathy*	1
*Hypertrophic cardiomyopathy*	1
*Arrhythmogenic dysplasia of the right ventricle*	2
*Non-compaction cardiomyopathy*	2
**Comorbid conditions**	***n***
Diabetes mellitus, *n*	89
Obesity, *n*	81
Anemia, *n*	43
Atrial fibrillation, *n*	49
**Smoking status**	
Current or ex-smoker, *n*	76
Nonsmoker, *n*	47
**Concomitant medications and devices**	***n* (%)**
Beta-blockers	114 (93)
Angiotensin converting enzyme inhibitors	75 (61)
Angiotensin receptor blockers	30 (24)
Valsartan + sacubitril	5 (4)
Mineralocorticoid receptor antagonists	97 (79)
Diuretics	89 (72)
Anticoagulants	57 (46)
Acetylsalicylic acid	80 (65)
Nitrates CRT-D	42 (34) 12 (10)

CAD—coronary artery disease, CRT-D—cardiac resynchronization treatment with defibrillator, LVEF—left ventricular ejection fraction. Values are indicated as number of patients *n* (%) or median (1st and 3rd quartiles).

**Table 2:** Comparison of patients with and without combined endpoint.

Parameters	Without Combined Endpoint (*n* = 47)	With Combined Endpoint (*n* = 76)	*p*-Value
***Clinical***			
Age, years	55 (47–58)	55 (46–59)	0.7
Sex (male/female), *n*	42/5	61/15	χ^2^ = 1.8; *p* = 0.2
FC, NYHA (II/III/IV)	24/18/5	22/34/20	χ^2^ = 7.6; *p* = 0.022
Smoking status, *n*	28	41	χ^2^ = 0.4; *p* = 0.53
BMI, kg/m^2^	26.3 (24; 33)	25.9 (22; 31)	0.44
Ischemic etiology, *n*	29	48	χ^2^ = 0.06; *p* = 0.8
LVEF Simpson, %	26 (20–29)	22 (16–27)	0.005
Epworth scale, points	5 (2–10)	6 (3–11)	0.7
CKD (eGFR < 60 mL/min/1.73 m^2^), *n*	11	24	χ^2^ = 1.2; *p* = 0.28
Biomarkers *			
NT-proBNP, pg/mL (0–125)	619 (401–676)	2464 (928–4955)	0.001
sST-2, ng/mL (<35)	33 (31–35)	27 (24–34)	0.5
CRP, mg/mL (<5)	3 (1.8–7.7)	3.2 (1.7–5.9)	0.1
Blood parameters *			
Uric acid, mcmol/L (210–420 male, 150–350 female)	510 (425–673)	480 (351–663)	0.21
eGFR (CKDEPI), mL/min/1.73 m^2^	72 (59–82)	79 (51–84)	0.95
Total cholesterol, mmol/L (<5.0)	5.0 (3.7–5.8)	3.6 (3–5.8)	0.14
HDL, mmol/L (>1.0 male >1.2 female)	0.9 (0.8–1.1)	1.1 (0.6–1.6)	0.4
LDL, mmol/L (<3.0)	3.2 (2.5–3.9)	2.3 (2.1–3.4)	0.5
***PSG parameters***			
AHI, episodes/h	26 (11–47)	12 (8–34)	0.9
OAI, episodes/h	0.6 (0–10)	0.7 (0.2–4)	0.1
CAI, episodes/h	3 (0.05–15)	0.6 (0–4)	0.4
MAI, episodes/h	7 (0–13)	0.7 (0–2.5)	0.4
HAI, episodes/h	11 (8–15)	10 (7–12)	0.088
ODI, episodes/h	29 (12–50)	15 (9–24)	0.3
SpO_2_Ave, %	93 (92–95)	94 (93–96)	0.4
SpO_2_Min, %	81 (77–88)	85 (79–87)	0.5
S_2_, %	51 (44–56)	43 (31–56)	0.007
WASO, min	20 (9–76)	108 (38–143)	0.003
Mean duration of apnea, sec.	22 (20–28)	23 (19–28)	0.97
Max duration of apnea, sec.	47 (35–76)	49 (35–70)	0.7
***Cardiopulmonary exercise testing***			
VO_2 peak_, mL/kg/min	18 (13–20)	15 (11–18)	0.003
VE/VCO_2_ slope	34 (31–42)	41 (36–56)	0.004

AHI—apnea–hypopnea index, CAI—central apnea index, CKD-EPI—Chronic Kidney Disease Epidemiology Collaboration, CRP—C-reactive protein, eGFR—estimated glomerular filtration rate, FC—functional class, HAI—hypopnea index, HDL—high-density lipoproteins, LDL—low-density lipoproteins, LVEF—left ventricular ejection fraction, MAI—mixed apnea index, NT-proBNP—the N-terminal prohormone of brain natriuretic peptide, OAI—obstructive apnea index, ODI—oxygen desaturation index, S_2_—duration of sleep stage 2, SpO_2_Ave and SpO_2_Min—average and minimum saturation O_2_, sST2—soluble form of suppression of tumorigenicity-2, TST—total sleep time, VE/VCO_2_ slope—the ventilator equivalent of CO_2_, VO_2_ peak—peak oxygen consumption, WASO—wake after sleep onset, sleep efficiency. *—normal values are indicated in brackets. Values are indicated as median (1st and 3rd quartiles).

**Table 3:** ROC-curve analysis of clinical, laboratory and instrumental parameters.

Parameter	AUC	SE	AS	Asymptotic 95% CI	
				Lower Bound	Upper Bound
Age	0.498	0.054	0.976	0.393	0.604
BMI	0.458	0.055	0.443	0.351	0.565
NTproBNP	0.888	0.054	0.001	0.782	0.994
CRP	0.602	0.053	0.067	0.497	0.707
eGFR (CKDEPI)	0.485	0.054	0.789	0.380	0.591
VO_2peak_	0.287	0.066	0.003	0.157	0.416
VO_2_/HR	0.426	0.073	0.329	0.282	0.570
VE/VCO_2_	0.709	0.073	0.004	0.566	0.852
LVEF	0.349	0.051	0.006	0.249	0.449
Sleep efficiency	0.447	0.059	0.373	0.331	0.562
AHI, episodes/h	0.488	0.058	0.835	0.375	0.602
TST	0.426	0.059	0.214	0.310	0.541
S_2_	0.339	0.055	0.007	0.232	0.446
WASO	0.824	0.080	0.004	0.668	0.980
SpO_2_Ave	0.550	0.057	0.379	0.439	0.661

AHI—apnea-hypopnea index, BMI—body mass index, CRP—C-reactive protein, CKD-EPI—Chronic Kidney Disease Epidemiology Collaboration, eGFR—estimated glomerular filtration rate, LVEF—left ventricular ejection fraction, NT-proBNP—the N-terminal prohormone of brain natriuretic peptide, S_2_—duration of sleep stage 2, SpO_2_Ave—average saturation O_2_, TST—total sleep time, VO_2_/HR (heart rate) oxygen pulse, VO_2_ peak—peak oxygen consumption, VE/VCO_2_ slope—the ventilator equivalent of CO_2_. AS—asymptotic significance, AUC—area under curve, CI—confidence interval, SE—standard error.

## Data Availability

Data is contained within the article.
